# Predicting local persistence/recurrence after radiation therapy for head and neck cancer from PET/CT using a multi-objective, multi-classifier radiomics model

**DOI:** 10.3389/fonc.2022.955712

**Published:** 2022-09-29

**Authors:** Qiongwen Zhang, Kai Wang, Zhiguo Zhou, Genggeng Qin, Lei Wang, Ping Li, David Sher, Steve Jiang, Jing Wang

**Affiliations:** ^1^ Department of Head and Neck Oncology, Department of Radiation Oncology, Cancer Center, and State Key Laboratory of Biotherapy, West China Hospital of Sichuan University, Chengdu, China; ^2^ Department of Radiation Oncology, University of Texas Southwestern Medical Center, Dallas, TX, United States; ^3^ Peter O’Donnell Jr. School of Public Health, University of Texas Southwestern Medical Center, Dallas, TX, United States

**Keywords:** head and neck squamous cell cancers, radiotherapy, treatment outcome prediction, radiomics, local persistence and recurrence

## Abstract

**Objectives:**

Accurate identifying head and neck squamous cell cancer (HNSCC) patients at high risk of local persistence/recurrence (P/R) is of importance for personalized patient management. Here we developed a multi-objective, multi-classifier radiomics model for early HNSCC local P/R prediction based on post-treatment PET/CT scans and clinical data.

**Materials and methods:**

We retrospectively identified 328 individuals (69 patients have local P/R) with HNSCC treated with definitive radiation therapy at our institution. The median follow-up from treatment completion to the first surveillance PET/CT imaging was 114 days (range: 82-159 days). Post-treatment PET/CT scans were reviewed and contoured for all patients. For each imaging modality, we extracted 257 radiomic features to build a multi-objective radiomics model with sensitivity, specificity, and feature sparsity as objectives for model training. Multiple representative classifiers were combined to construct the predictive model. The output probabilities of models built with features from various modalities were fused together to make the final prediction.

**Results:**

We built and evaluated three single-modality models and two multi-modality models. The combination of PET, CT, and clinical data in the multi-objective, multi-classifier radiomics model trended towards the best prediction performance, with a sensitivity of 93%, specificity of 83%, accuracy of 85%, and AUC of 0.94.

**Conclusion:**

Our study demonstrates the feasibility of employing a multi-objective, multi-classifier radiomics model with PET/CT radiomic features and clinical data to predict outcomes for patients with HNSCC after radiation therapy. The proposed prediction model shows the potential to detect cancer local P/R early after radiation therapy.

## Introduction

In the United States, head and neck squamous cell cancers (HNSCC) represent a substantial number of cancers, with an estimated 53,000 new cases per year and 10,800 deaths from the disease (1). Patients with HNSCC often require radiation therapy as part of their treatment. Radiation can be recommended as a definitive treatment with or without chemotherapy, as an adjuvant treatment after surgery, or in combination with other treatment modalities (2, 3). However, even when treated with curative intent, 25-50% of patients with HNSCC will experience recurrence, predominantly within the first three years after treatment (4, 5). Therefore, accurate and early prediction/detection of tumor persistence/recurrence (P/R) would be valuable for making treatment decisions after radiotherapy. For patients at high risk for recurrence, intensified treatments or more frequent post-treatment surveillance imaging are needed. On the other hand, for patients at low risk for recurrence, unnecessary repeated surveillance scans may be avoided (6).

Fluorodeoxyglucose (FDG) PET/CT is routinely used for post-radiation treatment surveillance to detect cancer P/R in HNSCC, as it provides both anatomic and metabolic information (7, 8). However, the oncologic use of PET/CT in the post-treatment assessment presents formidable challenges due to treatment-related inflammation, which involves the increased FDG uptake and ultimately lead to false-positive interpretation (7, 9, 10). For instance, Mester et al. showed that more than one quarter of 1,134 FDG PET/CT reports contained incidental foci of begin FDG uptake and the majority were caused by inflammatory processes (10). Thus, it is recommended that PET/CT be performed at least 12 weeks after treatment completion to minimize inflammatory FDG uptake (8, 11). However, in a meta studies including 24 studies (2627 patients), assessment of PET/CT (>3 months) in detecting local failure demonstrated a sensitivity of 87% and a specificity of 93% and more tools are needed to improve the detection of local failure (12).

Radiomics involves advanced imaging analysis techniques that offer a possibility for differentiating intratumoral heterogeneity and observing a patient’s response to treatment by extracting quantitative features from radiological images such as CT, PET, MRI, or PET/CT (13, 14). These features, including some not easily visible or quantifiable upon visual inspection, can be used to build models for exploring predictive information from radiological images (15–18). For HNSCC treatment outcome prediction, Vallieres et al. developed a set of random forest method based radiomics model to predict locoregional failure (LF) and distant metastases using PET/CT data collected before treatment (19). With a multi-institution dataset, they obtained an area under the receiver operating characteristic curve (AUC) of 0.86 for DM and 0.69 for LF. Using the same dataset, we built a multi-classifier, multi-objective and multi-modality model (mCOM) for pre-treatment HNSCC LF prediction (20). In the mCOM model, multiple classifiers were used to create the model; sensitivity and specificity were considered simultaneously as the objectives to guide the model construction. Both clinical features and radiomics features extracted from various modalities were used as model inputs. The optimal mCOM model achieved an AUC value of 0.77. In addition, Lv et al. used the same cohort to construct a multi-level multi-modality fusion radiomics model for prognostication of HNCSS, and similar performance was achieved (21). As more of HNCSS data are publicly available on The Cancer Imaging Archive (TCIA), they developed a context-aware saliency guided radiomics model using 806 HNSCC patients from 9 centers for survival time-event outcome prediction using pre-treatment imaging only (22). HEad and neCK TumOR (HECKTOR) challenge, organized as a satellite event of the International Conference on Medical Image Computing and Computer Assisted Intervention (MICCAI) from 2020, offered an opportunity for researchers accessing more HNSCC patient data and validation their HNSCC segmentation and outcome prediction model objectively (23). According to the overview of the challenge in 2021, radiomics based method played an important role in the submitted models (23).

Although radiomics is a hot topic for treatment outcome prediction of HNSCC, most of the works and datasets are focusing on pre-treatment outcome prediction. Here we hypothesize that a radiomic feature set extracted from PET/CT images of HNSCC primary tumors after radiation therapy correlates with patients’ local control and that a machine learning model can be trained by these features to predict patients’ local control after radiotherapy. The goal of this study was to develop and validate a multi-objective, multi-classifier radiomics model that can predict post-treatment local P/R in patients with HNSCC. As many clinical parameters such as patient age, tumor stage, primary site and HPV status have shown strong correlation to treatment outcome in different studies, we added several of these parameters as features for model training (19, 24, 25). In the multi-objective model, to select the most predictive feature set and to balance the model performance on prediction sensitivity and specificity, we optimized the sparsity of the selected radiomic feature set, the prediction sensitivity, and prediction specificity simultaneously through an immune algorithm. To improve the prediction robustness, we used three base classifiers—logistic regression (LR), discriminant analysis (DA), and support vector machine (SVM)—together to build the model. We developed the radiomics model with a training cohort and evaluated it on an independent validation cohort.

## Materials and methods

### Patients

**Figure 1:**
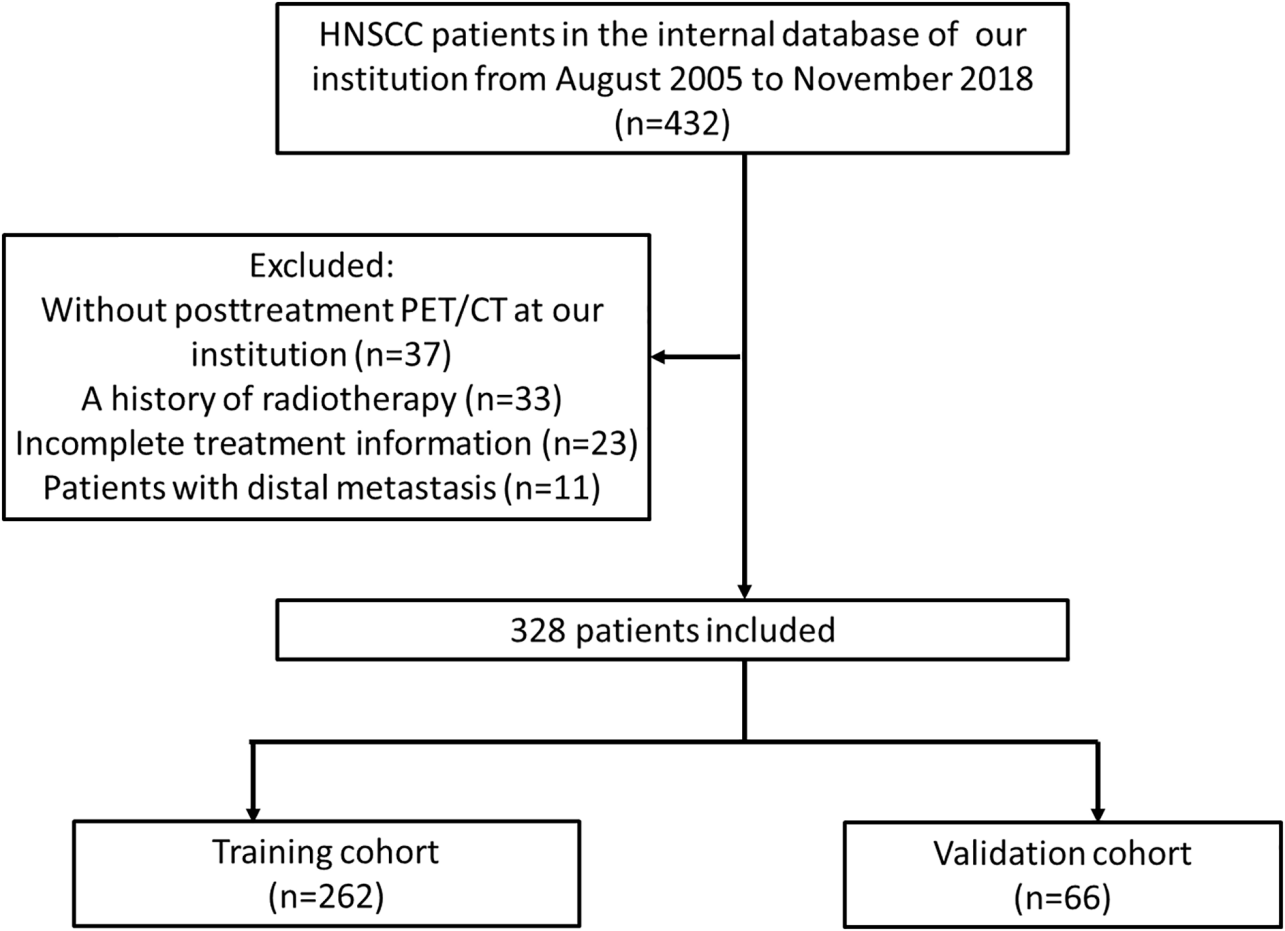
Diagram of the patients included in this study.

All investigations in this study were carried out in accordance with the guidelines and regulations of institutional review board (IRB). We originally included 432 patients with HNSCC diagnosed at our institution from August 2005 to November 2018. This institutional database contained information about the baseline features, therapy, and follow-up data. Patients with distant metastases, those who did not receive a PET/CT scan from the treating institution within six months after treatment, and those who had a history of radiotherapy were excluded. Thus, 328 patients were included in this study: 69 with tumor persistence/local recurrence and 259 without locoregional recurrence. All 69 cases who have locoregional recurrence were confirmed with biopsy (cytologic or histologic). 328 patients were divided into a training cohort (262 patients) and a validation cohort (66 patients) using baseline characteristics-based case-control matching with a 4:1 fashion (**Figure 1**). Baseline characteristics difference between training and validation cohorts were evaluated with t-test, Mann-Whitney U-test, and Fisher’s test for continuous data, ordinal data, and categorical data, respectively. We used SPSS version 26.0 (Armonk, NY: IBM Corp) to perform the matching, and P-value of <0.05 was set as significant.

### Treatments and clinical endpoints

Patients were treated with radiation therapy in different treatment modalities, including definitive radiation treatment (RT), concurrent chemoradiotherapy (CCRT), and surgery then RT alone or CCRT. Patients referred for re-irradiation were excluded. Cisplatin, paclitaxel, and cetuximab were combined with radiotherapy in different treatment regimens in CCRT. Each of these patients received a total radiation dose of 66-72 Gy in 2-2.2 Gy fractions to the gross tumor. Radiation treatments were performed according to a conventional (five fractions per week) schedule. And patients were followed up every 3-6 months for the first two years and every 6-12 months after the therapy, albeit the exact timing was subject to normal clinical variability. The follow-up practices included physical examination, blood test, PET/CT and considerable chest imaging, and a biopsy confirmation was commonly recommended if lesions were suspected of harboring malignancy. Recurrence was defined as re-emergence of the tumor after initial complete regression and was divided into local (i.e., treatment failure within the planning target volume or the zone of the primary tumor) and regional recurrence (i.e., failure in the neck lymph nodes). Recurrence was distinguished from a second primary tumor by considering pathological features, clinical features, and the location of the tumor in relation to radiotherapy (26). The median follow-up for this study is 61 months (range: 9-170 months).

### PET/CT image acquisition and image segmentation

Diagnostic post-treatment PET and CT scans were exported through the digital Picture Archiving Communication System (PACS, iSite Enterprise). The median follow-up time from treatment completion to PET/CT imaging was 114 days (range: 82-159 days). Gross tumor volume (GTV) was defined as areas suspicious for cancer local P/R after radiation therapy on PET/CT images, which included pretreatment GTVs and areas with a standardized uptake value (SUV) ≥ 2.5. A radiation oncologist and a nuclear medicine radiologist with more than six years of clinical experience contoured the GTVs on CT in Velocity AI (Varian Medical Systems, Palo Alto, CA, USA). Details about the PET/CT imaging protocol of the included patient scans are shown in **Supplementary Table S1**.

### Radiomic feature extraction

We extracted radiomic features from PET/CT images within the delineated GTV. We corrected GTV masks to cover soft-tissue-only areas by removing voxels whose Hounsfield units (HU) are outside of [-150, 180] on CT images. For PET images, we calculated the standardized uptake value (SUV) (27). For CT images, we kept their image values in raw HU format. Before feature extraction, we resampled the voxel spacing of all the images to an isotropic voxel size of 1.0 × 1.0 × 1.0 mm^3^ via bilinear interpolation to correct for the differences in voxel spacing and slice thicknesses between different scans.

We extracted 257 radiomic features for each imaging modality by using a MATLAB based open source radiomics toolbox (28). These radiomic features comprise eight geometry features, nine intensity features, and 240 gray-level co-occurrence matrices (GLCMs) based texture features. See the **Supplementary Material** for more detailed explanations of the extracted features and their mathematical definitions.

### Clinical feature selection

In addition to radiomic features, clinical characteristics such as patient age, tumor stage, primary site and HPV status may improve the performance of local P/R prediction models (29–33). Several prospective clinical trials and retrospective analyses have shown that HPV status is strongly associated with therapeutic response and survival for individuals with HNSCC in the oropharynx (29, 30, 32). Besides, patient age, tumor primary site, tumor T-stage, and N-stage were found contribute significantly to prediction HNSCC treatment response and overall survival (31). Therefore, in this study, we also collected clinical information to build the prediction model.

### Multi-objective, multi-classifier prediction model

**Figure 2:**
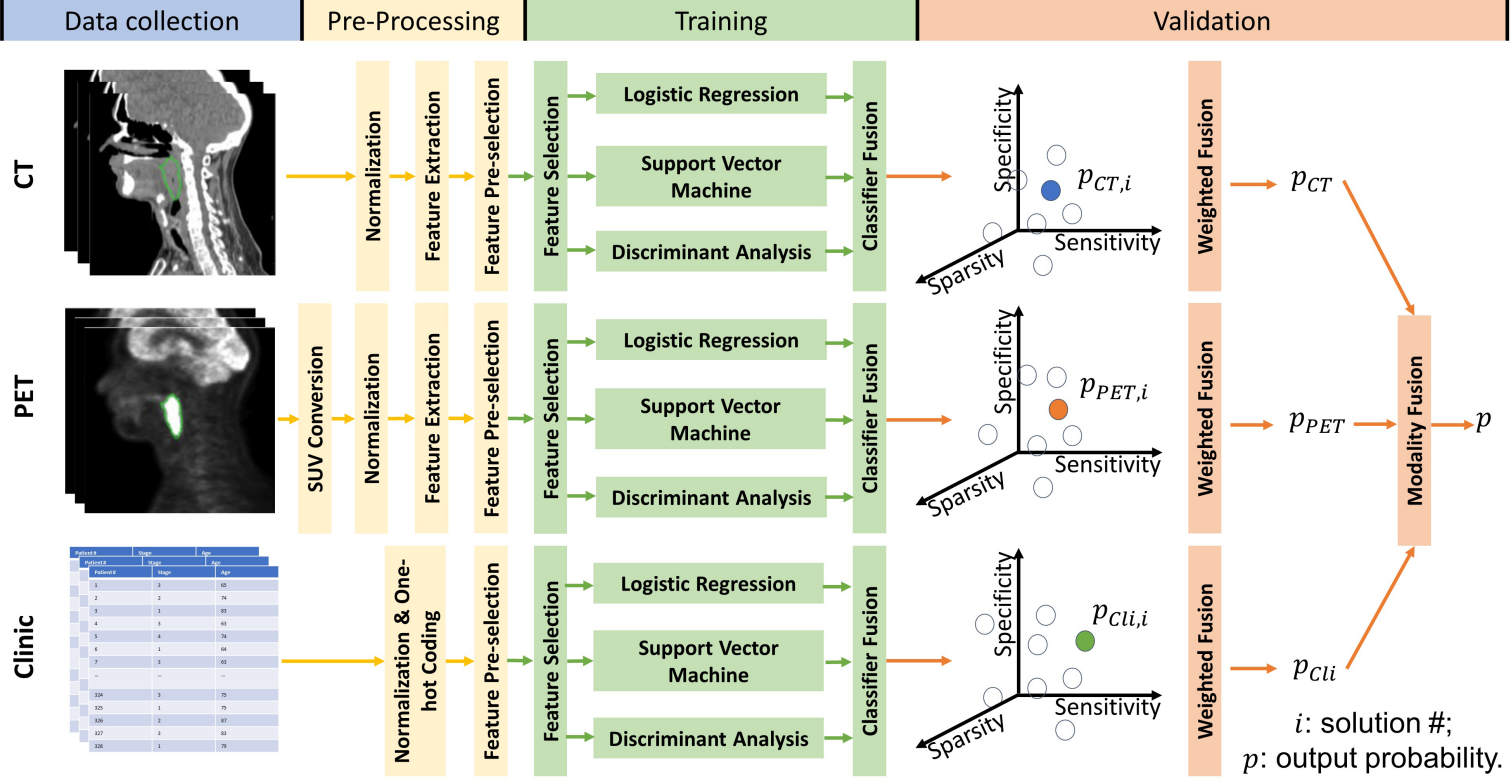
Workflow for the proposed multi-objective, multi-classifier, multi-modality radiomics model.

We created a multi-classifier, multi-objective, and multi-modality radiomics model to identify HNSCC patients who have a high risk of local P/R by fusing the output probabilities from separate predictive models built with features from different modalities: CT, PET, and clinical data. The training and prediction pipeline for the proposed radiomics model is illustrated in **Figure 2**.

Before model training, for both PET and CT radiomic features, we removed the redundant and less predictive radiomic features using the minimal-redundancy-maximal-relevance criterion (mRMR) method (34). The pre-selected feature group is of 50 features for each imaging modality. In the model training stage, we train the PET-radiomics, CT-radiomics, and clinical feature-based models separately. For each separate model, three representative classifiers—LR, SVM, and DA—were used together to improve the robustness of the prediction results. Model sensitivity, specificity and feature sparsity were optimized simultaneously to guide the model construction. Feature sparsity here is defined as one over the number of features selected through model training, where a higher value indicates that fewer features were selected to build the model, and the minimum number of features selected was set as 1. To create a model for each modality, we used an iterative multi-objective immune algorithm (IMIA) (35) to update the feature selection vector ƒ, hyperparameters of classifiers *β*, and weights of classifiers *ω* for classifier fusion according to the predictive performance on the training cohort. Different values and combinations of *ƒ*, *β* and *ω* result in different solutions, e.g., sub-models, and solutions in the pareto-optimal solution set were kept during model updating with IMIA. Each solution in the pareto-optimal solution set has either higher feature sparsity, higher sensitivity, or higher specificity on the training data than the other solutions in the pareto-optimal set. During model training, the probability output of solutions in the pareto-optimal solution set were weight-fused by using the evidential reasoning (ER) method to evaluate the training performance of the pareto-optimal solution set (36). The weight for each solution was determined by its performance on the training data. The more balanced the sensitivity and specificity, the higher the weight factor.

After the model performance on the training cohort converged or the number of model updating generations reached the maximum, which was set as 50 in our experiment, we calculated the weights of modalities for output probability fusion based on their training performances. Then, for a sample from the validation cohort, models built with clinical data, CT radiomic features, and PET radiomic features gave their prediction results separately. Finally, these prediction results were fused together *via* ER again to give a final prediction value. See the **Supplementary File** and recent studies (20, 37) for details about IMIA, classifier fusion, weighted fusion, and modality fusion. Of note, the proposed multi-classifier, multi-objective, and multi-modality radiomics model is based on our previous work (20), and we modified it by adding feature sparsity as an additional objective to further reduce the redundancy of the selected feature set during model training.

### Model performance evaluation

To evaluate the benefit of adding feature sparsity to the objective during model training, we compared the number of selected features and model performance of the multi-classifier, multi-objective radiomics models trained with and without feature sparsity objective for PET and CT separately. To evaluate the robustness of the multi-classifier model over single-classifier ones, we compared the prediction performance of PET and CT multi-classifier radiomics to their single-classifier ones (models built with LR, DA, and SVM separately). To illustrate the added prediction power of using multi-modality fusion strategy, we constructed five different multi-objective, multi-classifier prediction models for comparison. Three of them were single-modality models: clinical features model, CT radiomic features model and PET radiomic features model. The other two were multi-modality models: fused CT and PET radiomics model and fused Clinical, CT and PET features (CT+PET+Clinic) model.

We evaluated the final models by calculating the sensitivity, specificity, accuracy, and AUC of the prediction results on the validation cohort. We calculated the statistical differences between the receiver operating characteristic curves (ROCs) of different models by using the Delong test with a significance level of 0.05 (38). To evaluate the difference of local P/R free survival between the identified high- and low-risk patient groups by different models, we plotted Kaplan-Meier curves to show their locoregional recurrence free survival probabilities along follow-up, where log-rank test with significance level of 0.05 was used to compare the survival distributions. We also evaluated the prognostic value of the three-modality-fused model in predicting local and regional recurrence respectively to compare with the reported prediction performance of human experts in a meta-analysis. Model training, validation and performance analysis were performed using MATLAB 2020a (The MathWorks, Inc).

## Results

### Clinical characteristics of the patients

**Figure 3:**
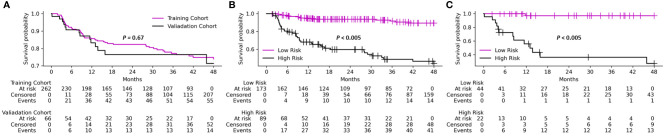
Kaplan-Meier analysis of local persistence/recurrence free survival on **(A)** training and validation cohorts, **(B)** identified low- and high-risk patient groups on training cohort, **(C)** identified low- and high-risk patient groups on validation cohort. P-values are calculated using log-rank test.

**Table 1** shows the patient characteristics for the training and validation cohorts. These cohorts had a median age of 66.4 (range 32-91) and were predominantly males (79.6%). The majority of the patients (98.8%) had a performance status (PS) score less than two, and oropharynx was the most common site of cancer (67.4%). We divided the therapeutic treatments toward HNSCC into four categories: CCRT (75.9%), RT alone (5.8%), surgery then RT alone (9.1%), and surgery then CCRT (9.1%). The training and validation cohorts did not differ significantly in terms of basic characteristics or recurrence rate (21.0% and 21.2%, respectively). Median follow-up time was 31 months (range 9-113 months) for the training cohort and 29 months (range 9-116 months) for the validation cohort. The Kaplan-Meier curves of local P/R free survival on training and validation cohort are shown in **Figure 3A**.

**Table 1:** Patient characteristics.

Characteristic	Training cohort	Validation cohort	P-value	Combined cohorts
**Patients (n)**	262	66		328
**Age (y)**				
Mean (SD)	66.0 (10.3)	68.1 (10.6)	0.148	66.4 (10.4)
Range	36-91	32-89		32-91
**Sex**			0.174	
Male	204 (77.9%)	57 (86.4%)		261 (79.6%)
Female	58 (22.1%)	9 (13.6%)		67 (20.4%)
**PS**			1	
Grade 0	177 (67.6%)	44 (66.7%)		221 (67.4%)
Grade 1	82 (31.3%)	21 (31.8%)		103 (31.4%)
Grade 2	3 (1.1%)	1 (1.5%)		4 (1.2%)
**Ethnicity**			0.301	
Caucasian	185 (70.6%)	42 (63.6%)		227 (69.2%)
African American	36 (13.7%)	11 (16.7%)		47 (14.3%)
Hispanic	23 (8.8%)	6 (9.1%)		29 (8.8%)
Asian	12 (4.6%)	2 (3.0%)		14 (4.3%)
Other	1 (0.4%)	1 (1.5%)		2 (0.6%)
Unknown	5 (1.9%)	4 (6.1%)		9 (2.7%)
**Smoking status**			0.825	
Never	89 (34.0%)	21 (31.8%)		110 (33.5%)
Former	124 (47.3%)	34 (51.5%)		158 (48.2%)
Current	49 (18.7%)	11 (16.7%)		60 (18.3%)
**Tumor site**			0.786	
Oropharynx	175 (66.8%)	46 (69.7%)		221 (67.4%)
Oral cavity	27 (10.3%)	4 (6.1%)		31 (9.5%)
Nasopharynx	50 (19.1%)	13 (19.7%)		63 (19.2%)
Larynx	10 (3.8%)	3 (4.5%)		13 (4.0%)
**T category**			0.915	
Tx	3 (1.1%)	0 (0%)		3 (0.9%)
T0	1 (0.4%)	0 (0%)		1 (0.3%)
T1	49 (18.7%)	10 (15.2%)		59 (18.0%)
T2	84 (32.1%)	23 (34.8%)		107 (32.6%)
T3	60 (22.9%)	19 (28.8%)		79 (24.1%)
T4	49 (18.7%)	11 (16.7%)		60 (18.3%)
Unknown	16 (6.1%)	3 (4.5%)		19 (5.8%)
**N category**			0.137	
N0	40 (15.3%)	17 (25.8%)		57 (17.4%)
N1	48 (18.3%)	7 (10.6%)		55 (16.8%)
N2	150 (57.3%)	35 (53.0%)		185 (56.4%)
N3	8 (3.1%)	4 (6.1%)		12 (3.7%)
Unknown	16 (6.1%)	3 (4.5%)		19 (5.8%)
**Grade**			0.691	
Low grade	9 (3.4%)	4 (6.1%)		13 (4.0%)
Intermediate grade	104 (39.7%)	28 (42.4%)		132 (40.2%)
High grade	94 (35.9%)	21 (31.8%)		115 (35.1%)
Unknown	55 (21.0%)	13 (19.7%)		68 (20.7%)
**HPV status**			0.689	
Negative	81 (30.9%)	19 (28.8%)		100 (30.5%)
Positive	61 (23.3%)	13 (19.7%)		75 (22.9%)
Unknown	120 (45.8%)	34 (51.5%)		154 (47.0%)
**Therapeutic combinations**			0.318	
Concurrent chemoradiotherapy (CCRT)	200 (76.3%)	49 (74.2%)		249 (75.9%)
Radiation alone	12 (4.6%)	7 (10.6%)		19 (5.8%)
Surgery then radiation alone	25 (9.5%)	5 (7.6%)		30 (9.1%)
Surgery then CCRT	25 (9.5%)	5 (7.6%)		30 (9.1%)
**Vital status**			0.151	
Alive	224 (85.5%)	51 (77.3%)		275 (83.8%)
Deceased	38 (14.5%)	15 (22.7%)		53 (16.2%)
**Local Control**			1	
Yes	207 (79.0%)	52 (78.8%)		259 (79.0%)
No	55 (21.0%)	14 (21.2%)		69 (21.0%)

### Model performance

The radiomics feature selection results and the validation performance are shown in **Supplementary Figures S1**, **S2**, **Table S2**, and **Table 2** when the models were trained with and without feature sparsity objective. As multiple objectives were used for model training, the final model for each modality is a fused model of different sub-models (solutions). Each of these solutions has different feature selection vector, hyperparameter, and classifier weight. Compared with models trained without the feature sparsity objective, the median number of selected features in each solution decreased from 13 to 5 for CT radiomics model, and 12 to 6 for PET radiomics model with feature sparsity serving as an additional objective. Meanwhile, the performance of models with feature sparsity objective numerically improved (**Table 2**). Of note, as most radiomics features in the pre-selected feature set (50 features) were less frequently selected when feature sparsity was used as an additional objective, the feature selection frequency for top features increased (**Figure S2**).

**Table 2:** Performance comparison of multi-classifier multi-objective radiomics models trained with and without feature sparsity (FS) as objective.

Modality	FS	Median feature number	Sensitivity	Specificity	Accuracy	AUC	P-value
CT	wo	13 (IQR: 12-15)	0.78	0.81	0.80	0.83	0.25
	w	5 (IQR: 4-8)	**0.86**	0.83	**0.83**	**0.85**	
PET	wo	12 (IQR: 8-16)	0.71	0.75	0.74	0.85	0.04
	w	6 (IQR: 3-8)	**0.93**	**0.83**	**0.85**	**0.90**	

The bold values indicate the best results of the related metrics for CT and PET radiomics models separately.

The performance comparison of radiomics models built with single classifier and multiple classifiers is shown in **Table 3**. Radiomics models built with the proposed multi-classifier strategy achieved numerically better performance for all evaluation metrics in both PET and CT radiomics models.

**Table 3:** Performance comparison of single classifier radiomics models and multi-classifier (MC) radiomics models.

Modality	Classifier	Sensitivity	Specificity	Accuracy	AUC	P-value
CT	LR	0.78	0.83	0.82	0.81	0.09
	DA	0.79	0.73	0.74	0.80	0.04
	SVM	0.71	0.81	0.79	0.82	0.11
	MC	**0.86**	**0.83**	**0.83**	**0.85**	—
PET	LR	0.93	0.75	0.79	0.88	0.43
	DA	0.71	0.81	0.79	0.87	0.16
	SVM	0.86	0.73	0.76	0.87	0.28
	MC	**0.93**	**0.83**	**0.85**	**0.90**	—

Classifiers comprising logistic regression (LR), discriminant analysis (DA), and support vector machine (SVM) were fused to construct the MC models. The bold values indicate the best results of the related metrics for CT and PET radiomics models separately.

**Figure 4:**
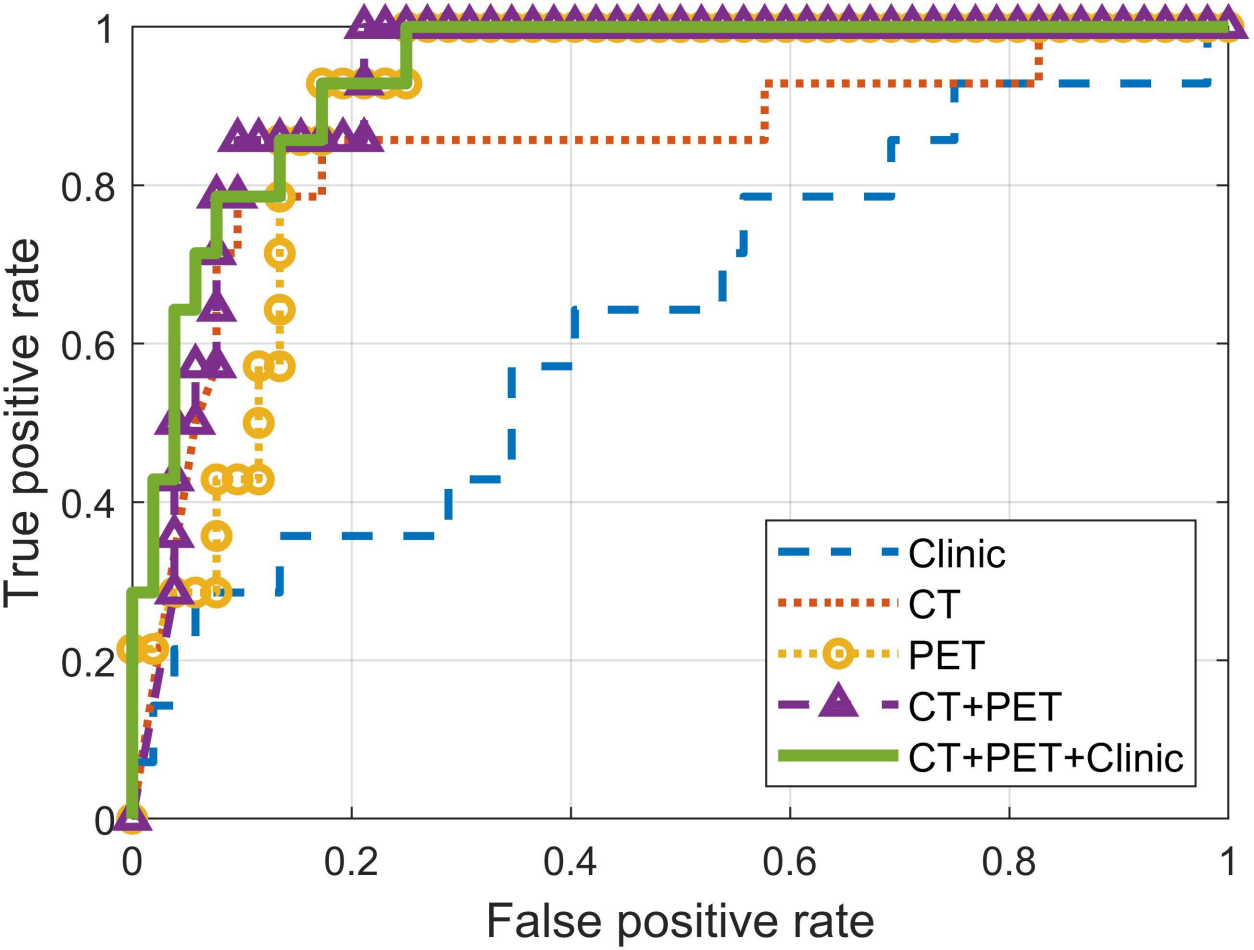
Receiver operating characteristic (ROC) curves of models built with features from different modalities.

The prediction performance of the separate single-modality models and the fused multi-modality models is summarized in **Table 4** and **Figure 4**. P-values in **Table 4** were calculated between the three-modality fused model and the other models. The fusion model of clinical features and post-treatment CT and PET radiomic features achieved the highest AUC (0.94) at predicting local P/R in the validation cohort. This was higher than both of the separate models, but only marginally higher than the fused CT and PET radiomics model. The CT+PET+Clinic model’s ROC did not differ significantly from that of the other models, except for the clinical feature–based model.

**Table 4:** Performance of multi-objective, multi-classifier models built with different combinations of modalities.

Modality	Sensitivity	Specificity	Accuracy	AUC	P-value
Clinic	0.64	0.60	0.61	0.63	<0.01
CT	0.86	0.83	0.83	0.85	0.08
PET	0.93	0.83	0.85	0.90	0.17
CT+PET	0.86	0.87	0.86	0.93	0.50
CT+PET+Clinic	**0.93**	**0.83**	**0.85**	**0.94**	—

Models were compared to the three-modality fusion model (CT+PET+Clinic) for calculating P-values. The bold values indicate the best results of the related metrics.

The Kaplan-Meier curves of local P/R free survival of differentiated low- and high-risk patient group using the CT+PET+Clinic model are shown in **Figures 3B, C** for training and validation cohort, respectively. The Kaplan-Meier curves of low- and high-risk patient groups identified by other models are shown in **Supplementary Figure S3**. Prediction probability value of 0.5 was used as the risk differentiation threshold for all the models. According to the results of log-rank test (**Figure 3**; **Figure S3**), the identified low-risk patient group using the CT+PET+Clinic model has significantly better local P/R free survival than the high-risk group in both training and validation cohorts. Meanwhile, the clinical feature model is the only model could not significantly identify high- and low-risk patients on the validation cohort (**Figure S3**).

In our dataset, 33 local failure and 25 regional failure were recorded in the training cohort, while the validation cohort patients had 11 local and 5 regional failure recorded. When probability value of 0.5 was used as the classification threshold, the three-modality-fused model predicted 23 patients in the validation cohort to have locoregional failure, in which 11 out of 11 local failure and 4 out of 5 regional failure was identified, however 9 patients were falsely predicted to be positive for locoregional failure. The corresponding sensitivities for local and regional failure are 100% and 80% respectively, while the overall specificity is 83%.

## Discussion

As radiation therapy is an important and potentially curative modality for HNSCC, a better local control is linked to improved disease-free survival and quality of life after treatment. In the present study, we developed a multi-objective, multi-classifier radiomics model using post-treatment PET/CT radiomics features and clinical data from HNSCC patients who had undergone radiotherapy, which could provide important information for predicting locoregional recurrence after radiotherapy. By adding feature sparsity as an additional objective to our previous published method, the median selected radiomics feature number decreased a half and better validation performance was achieved. The multi-classifier strategy produced more robust prediction performance than single classifier models. The fused post-treatment PET/CT radiomics model achieved promising performance (AUC=0.93) at predicting local P/R. The model that achieved the highest AUC value fused the output probabilities from all the single-modality models (AUC=0.94).

When evaluated the prognostic value of our best model in prediction local and regional failure separately, our model performed better sensitivities (sensitivity_local_ = 100% and sensitivity_regional_ = 80%) than radiologists’ diagnosis performance reported from a mate-analysis of 24 studies which included 2627 patients (sensitivity_local_ = 87% and sensitivity_regional_ = 79%), but lower specificity (specificity_model_ = 83% and specificity_radiologist_ = 93% to 95%) (12, 39). These results indicate our radiomics model was highly prognostic for locoregional tumor control with high sensitivity, which could help physicians detect tumor after radiotherapy and provide timely additional treatment to patients of high probabilities of tumor local P/R, however it might increase the risk for unnecessary treatment for false positive patients. Note that there is one difference between radiologists’ reading performance and our model prediction performance using post-treatment PET. Radiologists’ reading on PET is to make a diagnosis whether there is any recurrence at the time of post-treatment PET acquisition. In our model prediction, we do not necessarily identify recurrence on the post-treatment PET. Rather we are using the post-treatment PET to predict whether patients will have recurrence eventually, which could happen after the first post-treatment PET acquisition.

Because PET/CT is a preferred surveillance modality for HNSCC patients after radiotherapy, many efforts have been made to improve the accuracy, sensitivity, and specificity of PET/CT in predicting clinical outcomes or distinguishing locoregional recurrence from radiation-induced inflammation (40–43). Manca et al. reviewed the principles of quantitative PET/CT imaging as well as related technical issues in PET/CT’s clinical application and demonstrated that, although SUVs are widely used for prognostic purposes, there is no consensus criterion for response assessment across institutions (40). Choi et al. used dynamic contrast-enhanced (DCE) T1-weighted perfusion MRI to detect local tumor recurrence after definitive treatment for patients with head and neck cancer (41). Theyanalyzed the surveillance MRI images from 3, 6, and 12 months after the treatment. DCE-MRI showed higher sensitivity than conventional MRI for the characteristics that allowed accurate differentiation of the vascular microenvironment from scars. However, this study did not use early surveillance images, in which inflammation could affect the prediction outcome. Nevertheless, the developed model with post-treatment PET/CT acquired at the median time of 114 days after treatment completion can still be useful for personalized management. For example, patients at high-risk for recurrence may have more frequent follow-ups such that salvage surgery can be perform rapidly before tumors grow or spread further.

Our multi-objective, multi-classifier, and multi-modality model not only demonstrates the feasibility of predicting local P/R from PET/CT, but also indicates the potential to distinguish tumor sites from local inflammation. In clinical practice, physicians were generally unable to detect recurrence within 12 weeks. Because FDG uptake is not limited to cancer sites and may occur in a variety of inflammatory cells, sites of tumor recurrence and inflammation could be mistaken for each other due to their similar FDG levels. Therefore, it is recommended that PET/CT be conducted at least 12 weeks following the treatment completion to reduce the false-positive effects of inflammation. By optimizing training methods and including clinical inputs of PET/CT from before 12 weeks, the radiomics model may separate recurrence from local inflammation at earlier stages, which would shed light on the early prediction of tumor P/R from PET/CT imaging.

This study has some limitations that could be addressed in our future research. First, although the three-modality fused model trended towards better performance than the radiomics-only models, we could not prove that the fusion of clinical features and PET/CT radiomic features provides an additive benefit over PET and/or CT radiomic features. The limited patient population in this study may have inhibited our ability to detect the difference, but the three-modality fusion model is still promising and worth investigating prospectively in a larger cohort. Second, this prediction model was trained and validated on data from a single institution: all the patients included were consistent in treatment planning, follow-up management, and tumor recurrence/persistence assessment. Although our results are encouraging, a large multicenter cohort analysis to further validate the clinical applicability of this prediction model would be worthwhile, especially given the concern of robustness, reproducibility, and standardization to radiomics related study (44, 45). External validation of our model will be one of our future works when a post-treatment HNSCC dataset is available. Third, this study only included post-treatment PET/CT images in the model training. In the future, an advanced prediction model would be worth exploring when more data collected during HNSCC patient management (e.g., dosimetric factors, pre-treatment PET/CT images, on-board CBCT, MRI, and PET/MRI) become available.

In conclusion, we developed and validated a multi-objective, multi-classifier radiomics model for predicting local P/R after radiotherapy in patients with HNSCC by using post-treatment PET/CT and patients’ clinical data. This model was effective at predicting outcomes, which could provide clinicians with more information about potential recurrence for post-treatment tumors. The proposed strategy is worthy of further investigation in larger HNSCC cohorts and integrating features from other modalities.

## Data availability statement

The datasets presented in this article are not readily available because of institution regulations. Requests to access the datasets should be directed to jing.wang@utsouthwestern.edu.

## Author contributions

QZ and KW are co-first authors for this manuscript, and they made equal contributions to this study. All authors contributed to the article and approved the submitted version.

## Acknowledgments

This work was supported in part by the US National Institutes of Health R01CA251792 (JW and DS).

## Conflict of interest

The authors declare that the research was conducted in the absence of any commercial or financial relationships that could be construed as a potential conflict of interest.

## Publisher’s note

All claims expressed in this article are solely those of the authors and do not necessarily represent those of their affiliated organizations, or those of the publisher, the editors and the reviewers. Any product that may be evaluated in this article, or claim that may be made by its manufacturer, is not guaranteed or endorsed by the publisher.
