# Supplementary material for: Predicting local persistence/recurrence after radiation therapy for head and neck cancer from PET/CT using a multi-objective, multi-classifier radiomics model
Source: Front Oncol. 2022 Sep 29;12:955712. doi: 10.3389/fonc.2022.955712 (PMC9557184; doi:10.3389/fonc.2022.955712)
Supplement: Supplementary file 1 [file DataSheet_1.pdf]

Supplementary Material

1. Imaging protocol

Each of the included patients in this study has a post-treatment PET/CT scan with median follow-up time of 114 days (range: 82-159 days) from treatment completion to PET/CT imaging. As a single institution study, the imaging protocols are similar for all the patients. For PET imaging, most of the scans were acquired with SIEMENS 1094 machine (96.3%), with pixel spacing of 2.04 mm (97.6%), slices thickness of 3.00 mm (95.1%), model-based scatter correction method (100%), and PSF 3i21s image reconstruction method (93.0%). For CT of the FDG-PET/CT imaging, most of the scans were acquired with SIEMENS Biograph machine (97.6%), with pixel spacing of 0.98 mm (99.4%), slice thickness of 2.00 mm (84.1%), focal spot of 1.2 mm (95.7%), tube current of 100 mA (IQR: 91-110 mA), and exposure of 116 mAs (IQR: 107-136 mAs). We summarize the distribution of key parameters of imaging protocols for all the FDG-PET/CT scan in **Supplementary Table S1** below.

Supplementary Table S1: FDG-PET/CT imaging protocols

PET		CT	
Term	Number	Term	Number
**Manufacturer and Model Name**		**Manufacturer and Model Name**	
SIEMENS 1094	316 (96.3%)	SIEMENS Biograph	320 (97.6%)
CPS 1062	9 (2.7%)	SIEMENS Emotion	6 (1.8%)
CPS 1023	3 (1.0%)	GE MEDICAL SYSTEMS Discovery RX&ST	2 (0.6%)
**Pixel Spacing (mm)**		**Pixel Spacing (mm)**	
2.04	320 (97.6%)	0.98	326 (99.4%)
2.60	8 (2.4%)	1.37	2 (0.6%)
**Slice Thickness (mm)**		**Slice Thickness (mm)**	
3.00	312 (95.1%)	2.00	276 (84.1%)
2.43	12 (3.7%)	3.00	46 (14.0%)
3.38	4 (1.2%)	3.75	6 (1.8%)
**Scatter Correction Method**		**Focal Spot (mm)**	
Model-based	328 (100%)	1.2	314 (95.7%)
**Reconstruction Method**		0.95	13 (4.0%)
PSF 3i21s	305 (93.0%)	0.7	1 (0.3%)
OSEM2D 4i8s	16 (4.9%)	**Tube Current (mA)**	100 (IQR: 91-110)
OSEM 2i8s	7 (2.1%)	**Exposure (mAs)**	116 (IQR: 107-136)

2. Radiomics feature extraction

We extracted 257 radiomic features comprise 8 geometry features, 9 intensity features, and 240 texture features. Geometry features were GTV volume, major diameter, minor diameter, eccentricity, elongation, orientation, bounding box volume, and perimeter. Intensity features were minimum, maximum, mean, standard deviation, sum, median, skewness, kurtosis, and variance of image intensity within GTV. Texture features include energy, entropy, correlation, contrast, texture variance, sum-mean, inertia, cluster shade, cluster prominence, homogeneity, max-probability, and inverse variance. These texture features are based on 3D gray-level co-occurrence matrices (GLCMs) generated with four distinct voxel distances (1 mm, 2 mm, 3 mm and 4 mm) and five different gray levels (8, 16, 32, 64 and 128).

The formulas of the used GLCM features are listed as below.

A GLCM of size $N_g \times N_g$ (=64 x 64) is denoted with $\boldsymbol{P}$ and describes textural information of given ROI. The $\boldsymbol{P}(i, j)$ is defined as the number of times a voxels of intensity i and the voxel in a distance δ of intensity j.

The distance vector δ is from $\{(x, y, z)|x \in (-1,0,1)\ and\ y \in (-1,0,1)\ and\ z \in (-1,0,1)\ and\ (x, y, z) \neq (0,0,0)\}$. To account for discretization length differences, neighbors at a distance of 1, $\sqrt{2}$, $\sqrt{3}$ were given a weight of 1, $1/\sqrt{2}$, $1/\sqrt{3}$, respectively.

- $p(i,j) = P(i,j)/\sum P(i,j)$
- μ = mean of $p(i,j)$
- μ_x = mean of $p_x(i)$
- μ_y = mean of $p_y(j)$
- σ = standard deviation of $p(i,j)$
- $p_{x+y}(k) = \sum_i^{N_g} \sum_j^{N_g} p(i,j),\ i + j = k$
- $p_{x-y}(k) = \sum_i^{N_g} \sum_j^{N_g} p(i,j),\ |i - j| = k$

1) Energy

$$energy = \sum_{i=1}^{N_g} \sum_{j=1}^{N_g} p(i,j)^2$$

2) Entropy

$$entropy = -\sum_{i=1}^{N_g} \sum_{j=1}^{N_g} \log_2[P(i,j)] \times p(i,j)$$

3) Correlation

$$correlation = \frac{1}{\sigma} \sum_{i=1}^{N_g} \sum_{j=1}^{N_g} (i - \mu)(j - \mu)\, p(i,j)$$

4) Contrast

$$contrast = \sum_{k=1}^{N_g} k^2 \left\{ \sum_{i=1}^{N_g} \sum_{j=1}^{N_g} p(i,j) \quad |i - j| = k \right\}$$

5) Variance

$$variance = \frac{1}{2{N_g}^2} \sum_{i=1}^{N_g} \sum_{j=1}^{N_g} [(i - \mu)^2 p(i,j) + (j - \mu)^2 p(i,j)]$$

6) Sum-Mean

$$sum\ mean = \frac{1}{2{N_g}^2} \sum_{i=1}^{N_g} \sum_{j=1}^{N_g} (i + j) p(i,j)$$

7) Intertia

$$contrast = \sum_{i=1}^{N_g} \sum_{j=1}^{N_g} (i - j)^2\, p(i,j)$$

8) Cluster Shade

$$cluster\ shade = \sum_{i=1}^{N_g}\sum_{j=1}^{N_g}\left(i + j - \mu_x - \mu_y\right)^3 p(i,j)$$

9) Cluster Tendency

$$cluster\ tendency = \sum_{i=1}^{N_g}\sum_{j=1}^{N_g}\left(i + j - \mu_x - \mu_y\right)^2 p(i,j)$$

10) Homogeneity

$$homogeneity = \sum_{i=1}^{N_g}\sum_{j=1}^{N_g}\frac{p(i,j)}{1 + |i - j|}$$

11) Max-Probability

$$max\ probability = \max_{i,j} p(i,j)$$

12) Inverse Variance

$$inverse\ variance = \sum_{i=1}^{N_g}\sum_{j=1}^{N_g}\frac{p(i,j)}{1 + (i - j)^2}$$

3. Clinical features

In addition to radiomic features, clinical characteristics such as patient age, tumor stage, primary site and HPV status may improve the performance of local P/R prediction models. Several prospective clinical trials and retrospective analyses have shown that HPV status is strongly associated with therapeutic response and survival for individuals with HNSCC in the oropharynx. Besides, patient age, tumor primary site, tumor T-stage, and N-stage were found contribute significantly to prediction HNSCC treatment response and overall survival. Therefore, in this study, we collected gender, age, HPV status, T-stage, N-stage, primary tumor site, and treatment paradigm for constructing the clinical model. The full list of clinical features is shown below.

1) Gender: binary feature, 1 for male, 0 for female.
2) Age: numerical feature, value is age in year.
3) Oropharynx: binary feature, 1 corresponding to primary tumor in oropharynx, 0 corresponding to not.
4) Nasopharynx: binary feature, 1 corresponding to primary tumor in nasopharynx, 0 corresponding to not.
5) Hypopharynx: binary feature, 1 corresponding to primary tumor in hypopharynx, 0 corresponding to not.
6) Larynx: binary feature, 1 corresponding to primary tumor in larynx, 0 corresponding to not.
7) T-Stage: ordinal feature, 0 = TX, 1 = T0, 2 = T1, 3 = T2, 4 = T3, 5 = T4.
8) N-Stage: ordinal feature, 0 = NX, 1 = N0, 2 = N1, 3 = N2, 4 = N3.
9) HPV-Status: binary feature, 1 = HPV positive, 0 = unknown or HPV negative.

10) No NPV- Status: binary feature, 1 = HPV status unknow, 0 = HPV status recorded.
11) RT or CCRT: binary feature, 1 = RT alone, 0 = CCRT.
12) Surgery: binary feature, 1 = surgery, 0 = no surgery.

4. Overview of the multi-classifier multi-objective multi-modality radiomics model

In the multi-classifier multi-objective multi-modality radiomics model, solutions are the basic predictive components that need to be trained and updated during model training, and some of them will be selected and used for prediction in testing. In this work, a solution is defined as the integration of different types of classifiers. These classifiers are trained with the same selected feature set, and their output probabilities are fused into a single value according to weighting factors to form one feasible solution. For a given modality, a solution is denoted by $\theta = \{f, \beta, w\}$, where f denotes a binary feature selection vector (value of 1 refers to selecting the corresponding feature for training and evaluating the solution, value of 0 refers to rejecting the corresponding feature), β represents the parameters (including hyperparameters) of all the classifiers used in the solution, and w is the weighting factor of different classifiers for the classifier fusion. Different feasible solutions can be generated by changing the feature selection vector f, the hyperparameters of classifiers in β, or the weighting factor w.

The implementation of the proposed model consists of a model training stage and a testing stage. The former uses training and validation data to generate a Pareto-optimal solution set, and the latter fuses the output probabilities of solutions in the Pareto-optimal solution set for testing samples and make the final prediction. In the training stage, after the solution set is initialized, we use iterative multi-objective immune algorithm (IMIA) to optimize the model through iterative feature selection, classifier parameter training, evidential reasoning (ER)-based fusion of output probabilities of classifiers, and Pareto-optimal solution set updating. After the model is well trained, the final output probabilities of testing samples are calculated in the testing stage by a two-step automatic weighted ER fusion method, which consists of weighted solution fusion and modality fusion. The output probabilities of the Pareto-optimal solution set for each modality are fused first to calculate the output probability of that modality in weighted solution fusion, then the output probabilities of all the modalities are fused to calculate the final output probability in modality fusion.

5. Multiple objects in model training

Since the aim of outcome prediction is not only to obtain results with high accuracy, but also to get more reliable results with both high sensitivity and high specificity, a multi-objective model is desirable. To obtain a clinically desirable model, we simultaneously consider sensitivity, specificity, and feature sparsity as the objective functions for each modality, that is:

$$g = \max_{\theta}(g_{sen}, g_{spe}, g_{fea}) \tag{1}$$

g_{sen}, g_{spe} and g_{fea} are defined as:

$$g_{sen} = \frac{\mathrm{TP}}{\mathrm{TP} + \mathrm{FN}} \tag{2}$$

$$g_{spe} = \frac{\mathrm{TN}}{\mathrm{TN} + \mathrm{FP}} \tag{3}$$

$$g_{fea} = \frac{1}{\mathrm{SUM}(f)} \tag{4}$$

where TP is the number of true positives, TN is the number of true negatives, FP is the number of false positives, and FN is the number of false negatives; 0.5 is used as the threshold for determining the predicted label according to the output probability. The goal of the proposed model is to maximize g_{sen}, g_{spe} and g_{fea} simultaneously to obtain the Pareto-optimal solution set.

6. Iterative multi-objective immune algorithm (IMIA) for model training

IMIA consists of six steps: initialization, cloning, mutation, deletion, solution update and termination. In the initialization step, solution set S is randomly initialized as S_0, and $S_0 = \{\theta_1, \dots, \theta_{I_0}\}$, where I_0 is the number of solutions at the beginning of model training. Each individual solution $\theta_i, i = 1, 2, \dots, I_0$, is defined as a group of parameters comprising feature selection vector f_i, classifier parameter vector β_i and weighting factor vector w_i. f_i is a binary vector: a value of "1" indicates that the corresponding feature has been selected, while "0" indicates that it has not. β_i is the vector containing all the parameters, including hyperparameters, of different classifiers, and w_i is the weights used in classifier fusion to fuse the output probabilities of multiple classifiers into a single probability value. After initialization, the first generation of solution sets for different modalities can be trained using features from training samples. A validation set is then used to evaluate all the solutions, and their performance—as measured by sensitivity, specificity, and AUC—is recorded and used as the basis for the solution cloning, mutation, and deletion operations in the next generation.

We use proportional cloning for solution cloning. An solution with a larger crowding-distance is reproduced more times, and the clonal time q_i for each solution is calculated as: $q_i = \left\lceil n_c \times \frac{\delta(\theta_i, S_t)}{\sum_{j=1}^{I} \delta(\theta_j, S_t)} \right\rceil$, where n_c is the expectant value of the clonal population, S_t is the solution set at generation t ($t = 1, 2, \dots, T$ and T is the maximal number of generations), and $\delta(\theta_i, S_t)$ represents the crowding distance.

The mutation operation will be performed on the cloned population C_t. For each parameter in the individual solution, a random mutation probability (MP_i) will be generated. If MP_i is larger than a general mutation probability (GMP_i), the mutation will occur. The mutated solution set is denoted by M_t. S_t and M_t are combined to form a new solution set F_t.

When the same solutions are generated in the solution set, the diversity of the individuals in a population will be reduced. We perform the deleting operation to ensure that all the solutions in the solution set are different after executing the clonal and mutation operations. If there are duplicated solutions in the new population F_t, we will only keep the unique one and delete other duplicated solutions. If the size of F_t is less than I_0, cloning and mutation should be used to generate more mutated individuals.

After deleting the duplicated solutions in the new solution set F_t, if the generation number reaches T, we select the Pareto-optimal solution set from F_T and use it for model testing. Otherwise, we get $S_{t+1} = F_t$. For each solution in S_{t+1} we retrain the classifiers using training data and recording their performance on validation data and repeat the cloning, mutation, and deletion operations.

7. Evidential reasoning

Among multi-modality radiomics methods, early integration and late integration are two typical approaches to fuse information provided by different modalities. Early integration methods directly concatenate all the features together to train a single model, while late integration methods construct separate classifiers using features from different modalities and combine the outputs of these classifiers by using fusion techniques. In our work, we used evidential reasoning (ER)[21-23] as the fusion method for late integration of the output probabilities of individual classifiers or models. In the training stage, for each solution, ER fused the output probabilities of different classifiers; we defined this process as classifier fusion. In the testing stage, ER first fused the output probabilities of the Pareto-optimal solution set of each modality into one probability, then combined the probabilities from different modalities into one final output probability; we referred to these processes as solution fusion and modality fusion, respectively.

Take classifier fusion for a solution built with CT radiomics features as an example. Assume that, at generation t, $t = 1, 2, \dots, T$ (where T is the number of the final generation), for a given feature selection vector $f_{CT,i}^{t}$, $i = 1, 2, \dots, I$ (where I is the number of solutions at generation i), the probability vector $p = \{p_{CT,1,i}^{t}, p_{CT,2,i}^{t}, p_{CT,3,i}^{t}\}$ represents the output LR probabilities from different classifiers C_1, C_2, C_3, and the

parameters of these three classifiers are set as the elements in parameter vector $\beta^t_{CT,i} = \{\beta^t_{CT,1,i}, \beta^t_{CT,2,i}, \beta^t_{CT,3,i}\}$, separately. In this study, we chose three classifiers that are commonly used in radiomics: support vector machine (SVM), discriminant analysis (DA), and logistic regression (LR). Given the corresponding weight vector of this solution, $w^t_{CT,i} = \{w^t_{CT,1,i}, w^t_{CT,2,i}, w^t_{CT,3,i}\}$, which satisfies $\sum_{c=1}^{3} w^t_{CT,c,i} = 1$, $0 \le w^t_{CT,c,i} \le 1$, the final output $p^t_{CT,i}$ is obtained through the following equations:

$$p^t_{CT,i} = \frac{\mu \times [\prod_{c=1}^{3}(w^t_{CT,c,i} p^t_{CT,c,i} + 1 - w^t_{CT,c,i}) - \prod_{c=1}^{3}(1 - w^t_{CT,c,i})]}{1 - \mu \times [\prod_{c=1}^{C}(1 - w^t_{CT,c,i})]}, \tag{5}$$

where μ is calculated as:

$$\mu = \left[\prod_{c=1}^{3}\left(w^t_{CT,c,i} p^t_{CT,c,i} + 1 - w^t_{CT,c,i}\right) + \prod_{c=1}^{3}\left(1 - w^t_{CT,c,i} p^t_{CT,c,i}\right) - \prod_{c=1}^{3}\left(1 - w^t_{CT,c,i}\right)\right]^{-1}. \tag{6}$$

In the training stage, the weighting factor w for classifier fusion is generated and iteratively updated by IMIA. In the testing stage, weighting factor w' for solution fusion and modality fusion is calculated according to a weighting function that can tune the balance between model sensitivity and specificity. In this study, we wish to get a balanced result, and we set the weight for solutions with extremely imbalanced sensitivity or specificity to zero. The weighting function is defined as:

$$w' = \begin{cases} \frac{g_{sen}}{g_{spe}} + \text{AUC} & \text{when } 0.5 \le \frac{g_{sen}}{g_{spe}} \le 1 \\ \frac{g_{spe}}{g_{sen}} + \text{AUC} & \text{when } 0.5 \le \frac{g_{spe}}{g_{sen}} \le 1. \\ 0 & \text{Other situations} \end{cases} \tag{7}$$

g_{sen}, g_{spe} and AUC denote the sensitivity, specificity, and AUC values, respectively, of each solution on the validation set, and w' is normalized into a unit vector thereafter. With the weighting vector automatically generated according to the performance on validation data, the ER fusion method can first fuse the output probabilities of solutions in the Pareto-optimal set for each modality in solution fusion, and then fuse the output probabilities of each modality into the final probability in modality fusion.

8. Model testing

After the IMIA optimization terminates, the Pareto-optimal solution set of each modality is well trained on the training and validation data. The solution for each modality S_m is the fusion of solutions in the corresponding Pareto-optimal set, which is denoted as:

$$S_m = \text{ER}\left(\theta_1, \ldots, \theta_{I_{max}}, w_{sol}\right), \tag{8}$$

where $\theta_1, \ldots, \theta_{I_{max}}$ are the solutions selected from the Pareto-optimal set after T generations, I_{max} is the number of these solutions, and w_{sol} is the automatically generated weighting factor vector for solution fusion; this vector is calculated by using Eq. (A.7) based on the validation performance of each solution. The final solution for the whole model is defined as:

$$S_{fin} = \text{ER}(S_1, \ldots, S_M, w_{mod}), \tag{9}$$

where $S_1, \ldots, S_M$ are the fused solutions for different modalities, M is the number of modalities (which is 3 in this study), and w_{mod} is the automatically generated weighting factor vector for modality fusion; this vector is calculated by using Eq. (A.7) based on the validation performance of each modality. For a testing sample, first, the features for each solution in S_m (m=1, 2, 3) are selected; second, each solution in S_m outputs a probability $p^T_{m,i}$ (i=1, 2,…, I_{max}) through its internal classifier fusion; third, the output probability of the solution set for each modality p_m is obtained through solution fusion of $p^T_{m,i}$, and p_m to yield the final output probability for each single-modality model; finally, modality fusion fuses the p_m of different modalities to calculate the final output probability of the multi-modality model, and the label can be determined then.

9. Selected features in the final model

As sensitivity, specificity, and feature sparsity were employed simultaneously as the objective in the model optimization process using IMIA, the final model for each modality contains a set of sub-models, i.e., Pareto-optimal solutions. Each of these solutions has its own feature selection vector and classifier parameters, achieving either higher sensitivity, higher specificity, or higher feature selection sparsity (less feature) than other solutions for the corresponding modality. In our experiment, the total number of Pareto-optimal solutions for clinical-based model, PET radiomics model, and CT radiomics model are 26, 30, and 30, respectively. And the average number of selected features in each solution for clinical-based model, PET radiomics model, and CT radiomics model are 4.0, 6.0, and 4.5, respectively. All the clinical features were selected at least once in the clinical model, 40 features were selected at least once in the PET radiomics model, and 40 features were selected at least once in the CT radiomics model. The distributions of selected feature numbers in each solution of radiomics models built with and without feature sparsity as objective are shown in Supplementary Figure S1, the feature selection frequency for the selected features (sorted) is shown in Supplementary Figure S2. The detail of most frequently selected features (top five features) for different modalities are summarized in Supplementary Table S2.

Supplementary Figure S1. Number of selected features in each solution of the PET radiomics models and CT radiomics models built with and without feature sparsity as objective.

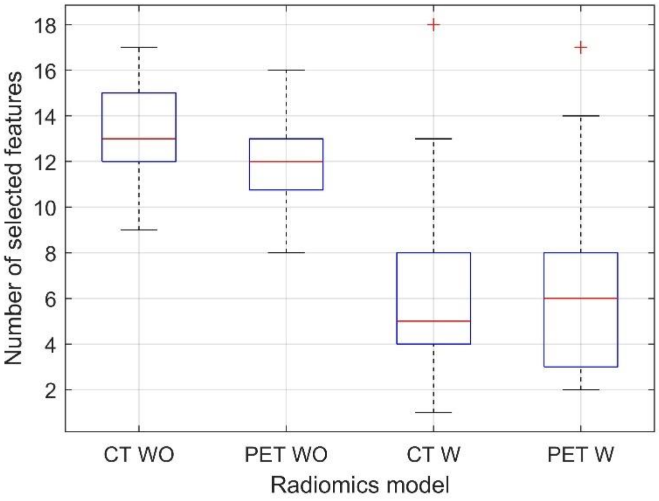

Supplementary Figure S2. Feature selection frequency in all the solutions of PET radiomics model and CT radiomics model, with and without feature sparsity as objective.

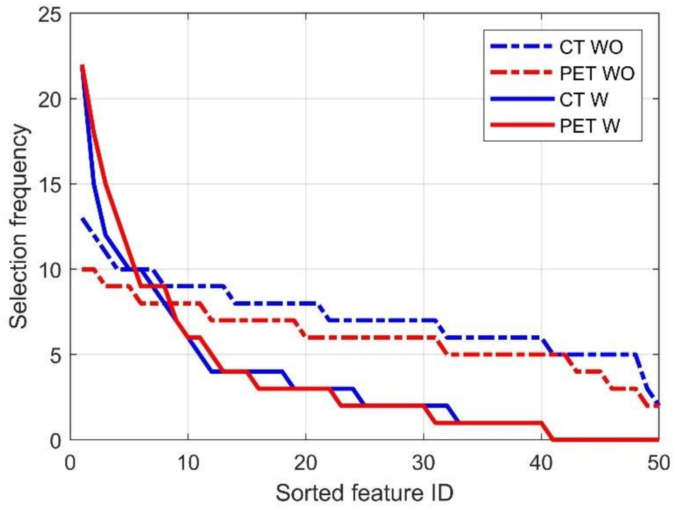

Supplementary Table S2. Most frequently selected ten features for PET radiomics model, CT radiomics model and clinical feature model.

PET Radiomics Model		CT Radiomics Model		Clinical Model	
**Feature Name**	**Selection Frequency**	**Feature Name**	**Selection Frequency**	**Feature Name**	**Selection Frequency**
Contrast-32-3	22	Eccentricity	22	T-Stage	16
Bounding box volume	18	Bounding box volume	15	HPV-Status	15
Eccentricity	15	Correlation-16-1	12	Surgery	11
Orientation	13	Intensity skewness	11	Male	9
Energy-8-4	11	Intensity kurtosis	10	Nasopharynx	8
Major diameter	9	ClusterTendency-128-3	10	Hypopharynx	8
Intensity minimum	9	SumMean-16-3	9	Age	8
Intensity standard deviation	9	SumMean-16-4	8	No HPV-Status	7
Contrast-16-3	7	Correlation-8-4	7	Oropharynx	6
ClusterShade-16-2	6	Major diameter	6	Larynx	6

* For radiomics features, the names were defined as FeatureName-GrayLevel-VoxelDistance.

10. Kaplan-Meier analysis

We plotted Kaplan-Meier curves of identified high- and low-risk patient groups with different prediction models for local P/R after radiotherapy, and we used log-rank test with significance level of 0.05 ($-\log_2 P$ of 4.32) to compare the survival distributions (**Supplementary Figure S3, Table S3**). Prediction probability value of 0.5 was used as the risk differentiation threshold for all the models. According to the results of log-rank test (**Figure S3, Table S3**), the identified low-risk patient group using all the prediction model has significantly better local P/R free survival than the high-risk group on both training and validation cohorts, except for the clinical feature model on the validation cohort.

Supplementary Table S3. Log-rank test P-value between the local persistence/recurrence free survival of identified low-risk patient group and high-risk patient group with different models.

Modality	$-\log_2 P$	
	Training Cohort	Validation Cohort
Clinic	26.68	2.99
CT	26.69	21.41
PET	47.48	27.25
CT+PET	29.92	25.71
CT+PET+Clinic	46.34	25.73

*As the P-values are too small here, we showed $-\log_2 P$ instead, and values larger than -log2(0.05) = 4.32 are viewed as significant.

Supplementary Figure S3. Kaplan-Meier analysis of local persistence/recurrence free survival on low- and high-risk patient groups of training and validation cohort identified by (A) clinical feature model, (B) CT radiomics model, (C) PET radiomics model, and (D) fused CT and PET radiomics model.

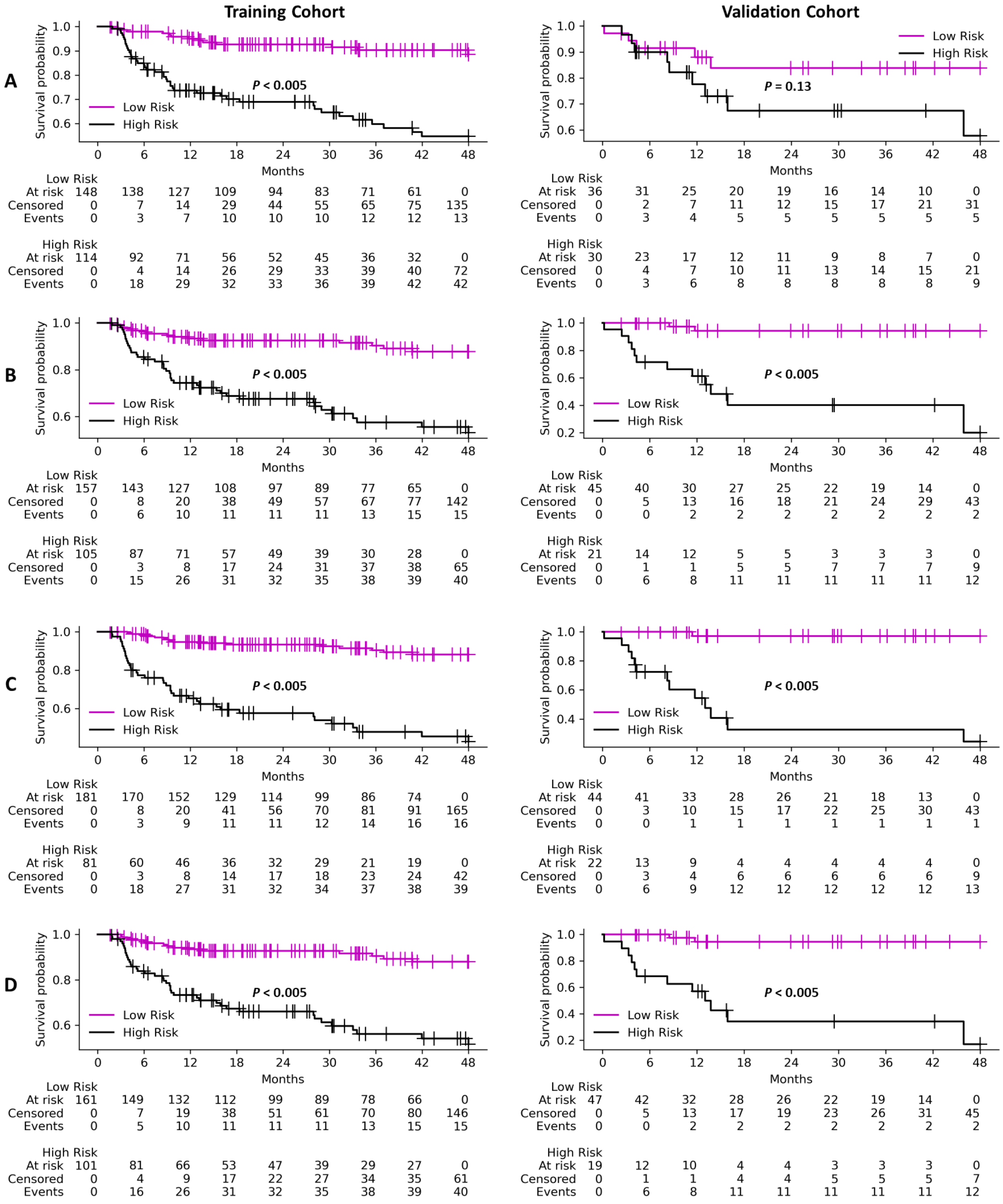
Training Cohort
Validation Cohort
A
B
C
D
Survival probability
Months
Low Risk
High Risk
P < 0.005
P = 0.13
A, Training Cohort:
Low Risk
At risk 148 138 127 109 94 83 71 61 0
Censored 0 7 14 29 44 55 65 75 135
Events 0 3 7 10 10 10 12 12 13
High Risk
At risk 114 92 71 56 52 45 36 32 0
Censored 0 4 14 26 29 33 39 40 72
Events 0 18 29 32 33 36 39 42 42
A, Validation Cohort:
Low Risk
At risk 36 31 25 20 19 16 14 10 0
Censored 0 2 7 11 12 15 17 21 31
Events 0 3 4 5 5 5 5 5 5
High Risk
At risk 30 23 17 12 11 9 8 7 0
Censored 0 4 7 10 11 13 14 15 21
Events 0 3 6 8 8 8 8 8 9
B, Training Cohort:
Low Risk
At risk 157 143 127 108 97 89 77 65 0
Censored 0 8 20 38 49 57 67 77 142
Events 0 6 10 11 11 11 13 15 15
High Risk
At risk 105 87 71 57 49 39 30 28 0
Censored 0 3 8 17 24 31 37 38 65
Events 0 15 26 31 32 35 38 39 40
B, Validation Cohort:
Low Risk
At risk 45 40 30 27 25 22 19 14 0
Censored 0 5 13 16 18 21 24 29 43
Events 0 0 2 2 2 2 2 2 2
High Risk
At risk 21 14 12 5 5 3 3 3 0
Censored 0 1 1 5 5 7 7 7 9
Events 0 6 8 11 11 11 11 11 12
C, Training Cohort:
Low Risk
At risk 181 170 152 129 114 99 86 74 0
Censored 0 8 20 41 56 70 81 91 165
Events 0 3 9 11 11 12 14 16 16
High Risk
At risk 81 60 46 36 32 29 21 19 0
Censored 0 3 8 14 17 18 23 24 42
Events 0 18 27 31 32 34 37 38 39
C, Validation Cohort:
Low Risk
At risk 44 41 33 28 26 21 18 13 0
Censored 0 3 10 15 17 22 25 30 43
Events 0 0 1 1 1 1 1 1 1
High Risk
At risk 22 13 9 4 4 4 4 4 0
Censored 0 3 4 6 6 6 6 6 9
Events 0 6 9 12 12 12 12 12 13
D, Training Cohort:
Low Risk
At risk 161 149 132 112 99 89 78 66 0
Censored 0 7 19 38 51 61 70 80 146
Events 0 5 10 11 11 11 13 15 15
High Risk
At risk 101 81 66 53 47 39 29 27 0
Censored 0 4 9 17 22 27 34 35 61
Events 0 16 26 31 32 35 38 39 40
D, Validation Cohort:
Low Risk
At risk 47 42 32 28 26 22 19 14 0
Censored 0 5 13 17 19 23 26 31 45
Events 0 0 2 2 2 2 2 2 2
High Risk
At risk 19 12 10 4 4 3 3 3 0
Censored 0 1 1 4 4 5 5 5 7
Events 0 6 8 11 11 11 11 11 12
